# Characterization of the Fiber Protein C-Terminal Domain from *Klebsiella pneumoniae* Phage KlebP_144 and Evaluation of Its Anti-Capsular Activity

**DOI:** 10.3390/ijms27093883

**Published:** 2026-04-27

**Authors:** Bogdana I. Kravchuk, Natalia N. Golosova, Ekaterina A. Kondakova, Yana A. Khlusevich, Vyacheslav I. Yakubovskij, Margarita I. Arisova, Yuliya N. Kozlova, Nina V. Tikunova, Andrey L. Matveev

**Affiliations:** Institute of Chemical Biology and Fundamental Medicine Siberian Branch of Russian Academy of Sciences, 630090 Novosibirsk, Russia; semali328@gmail.com (B.I.K.); n.golosova@alumni.nsu.ru (N.N.G.); e.kondakova1@g.nsu.ru (E.A.K.); khlusevichjana@mail.ru (Y.A.K.); yakubovskij97@xmail.ru (V.I.Y.); m.arisova@g.nsu.ru (M.I.A.); ulona79@mail.ru (Y.N.K.); tikunova@1bio.ru (N.V.T.)

**Keywords:** *Klebsiella pneumoniae*, bacteriophage depolymerase, capsular polysaccharide, biofilm degradation, carbohydrate-binding motif, antimicrobial resistance, structural analysis, *K. pneumoniae*

## Abstract

*Klebsiella pneumoniae*, a member of the ESKAPEE group of priority pathogens, has become one of the most challenging bacterial pathogens in modern clinical practice, largely due to its multidrug resistance and the immune-evasive effect of its capsular polysaccharide (CPS). Phage-encoded depolymerases, which selectively degrade the capsular polysaccharide, have emerged as promising antimicrobial agents capable of restoring bacterial susceptibility to both immune clearance and phage infection. The fragment corresponding to the C-terminal region of a putative depolymerase of bacteriophage KlebP_144, namely DepKP144ΔC, was cloned, expressed in *E. coli*, and purified using immobilized metal affinity chromatography. DepKP144ΔC displays an enzymatic activity against capsular polysaccharides of 100% K1 capsular-type strains and 85% K2 capsular-type strains, including classical and hypervirulent isolates. It was demonstrated that this protein is capable of inhibiting *K. pneumoniae* biofilm formation, but it is unable to disrupt mature biofilms. In vivo experiments using a murine *K. pneumoniae* infection model further confirmed its therapeutic potential: treatment with DepKP144ΔC improved survival rate in mice infected with K2-type *K. pneumoniae*, indicating significant attenuation of bacterial virulence. Therefore, these results demonstrate the potential role of the C-terminal domain of the bacteriophage KP144 tail-fiber protein in phage entry and show that its carbohydrate-recognition motifs possess enzymatic activity against the *Klebsiella* capsular polysaccharides.

## 1. Introduction

*Klebsiella pneumoniae* has become one of the most clinically significant bacterial pathogens in recent years, owing to its growing prevalence in hospital environments and its ability to cause severe infections in vulnerable patients [1,2]. As a member of the ESKAPEE group—pathogens recognized by the WHO as priority targets for the development of new antimicrobials—*K. pneumoniae* represents a critical global health concern. This opportunistic species is frequently implicated in pneumonia, bloodstream, and urinary tract infections, particularly among immunocompromised and critically ill patients [3]. The rapid expansion of multidrug-resistant and carbapenem-resistant isolates has intensified global concern, as treatment options for these infections are increasingly limited [4,5]. A key factor underlying the success of *K. pneumoniae* as a nosocomial pathogen is the presence of a thick capsular polysaccharide (CPS) layer that serves as both a mechanical and immunological barrier [6,7]. This capsule not only protects bacterial cells from phagocytosis and complement-mediated killing but also promotes surface adherence and persistence under antibiotic pressure, ultimately contributing to chronic infection and poor clinical outcomes [6].

The phage infection begins when the phage tail fibers or tail spikes identify and bind specific surface structures on the bacterial cell, initiating a cascade that led to injection of viral nucleic acid [8]. This molecular mechanism defines the narrow host range characteristic of most phages and determines the outcome of infection [9,10]. In encapsulated bacteria such as *K. pneumoniae*, successful adsorption is often hindered by the thick capsular polysaccharide that masks cell surface receptors. Many *Klebsiella* phages encode virion-associated depolymerases that selectively cleave capsular polysaccharides, exposing the underlying bacterial surface and facilitating phage adsorption to its receptor. Typically, the tail spike or tail fiber protein has depolymerase activity, ensuring that enzymatic degradation of the capsule occurs in concert with receptor engagement [11]. By degrading this major protective barrier of bacterial cells, the enzyme not only enables infection but also attenuates bacterial virulence [12]. This combination of structural specificity and enzymatic precision has driven growing interest in depolymerases as potential antimicrobial therapeutics, capable of disarming pathogenic bacteria without exerting the selective pressure associated with conventional antibiotics [13].

The role of depolymerases becomes particularly relevant in the context of biofilm-associated infections [14,15]. Biofilms formed by *K. pneumoniae* are highly structured microbial communities embedded in a dense extracellular polysaccharide matrix. This organization restricts antibiotic penetration and enhances resistance to host immune defenses, making biofilm-associated infections notoriously difficult to eliminate [16]. Depolymerases targeting both capsular and extracellular polysaccharides have shown strong antibiofilm activity, suggesting their potential utility in managing persistent infections, especially in immunocompromised patients or individuals with prolonged hospitalization [17].

Phage-encoded polysaccharide depolymerases are characterized by modular architecture with three domains [18]. In most cases, the N-terminal regions of these proteins are involved in their incorporation into the virion structure and ensure the correct spatial orientation of the enzyme on the phage surface. The central β-helical domain forms a trimer and is responsible for both the recognition and enzymatic degradation of Gram-negative bacterial exopolysaccharides [19]. The C-terminal domain exhibits substantially higher variability and is either associated with specific binding to capsular and extracellular polysaccharides or includes molecular chaperones for folding and trimerization [18]. These domains largely determine the substrate specificity of depolymerases and, consequently, their selectivity toward particular capsular types of *K. pneumoniae*.

In the present study, the C-terminal fragment of the depolymerase encoded by *K. pneumoniae* phage KlebP_144, designated DepKP144ΔC, was selected as the primary object of analysis. To test this assumption, DepKP144ΔC was subjected to comprehensive characterization, including in silico prediction of its domain organization and three-dimensional structure, as well as phylogenetic analysis aimed at assessing its evolutionary relationships with depolymerases from other *Klebsiella*-specific bacteriophages and identifying possible links between structural features and capsular specificity. The functional properties of DepKP144ΔC were subsequently evaluated using a combination of in vitro assays and an in vivo infection model. Particular emphasis was placed on examining its activity against biofilm-associated forms of *K. pneumoniae*, given the well-established role of biofilms in infection persistence and reduced susceptibility to antimicrobial treatment.

## 2. Results

### 2.1. In Silico Characterization of the C-Terminal Domain of Depolymerase DepKP144

The C-terminal domain of DepKP144 (DepKP144ΔC) comprises 170 amino acids with a predicted isoelectric point (pI) of 4.88 and a molecular weight of approximately 18 kDa. Domain analysis was performed using the InterPro software package v.108 (https://www.ebi.ac.uk/interpro/search/sequence/, accessed on 5 February 2026). The results indicate that DepKP144 belongs to the Galactose-binding-like domain superfamily (IPR008979). Its central region (residues 20–169) exhibits similarity to the Galactose-binding domain-like fold (G3DSA:2.60.120.260).

**Figure 1:**
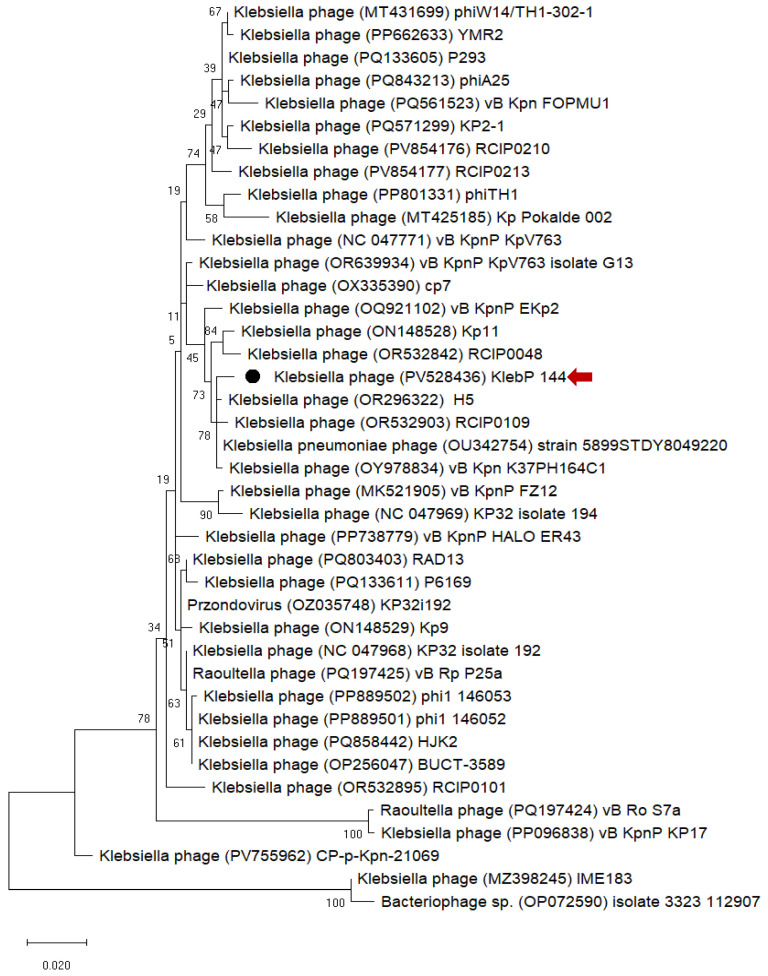
Phylogenetic analysis of the C-terminal depolymerase fragment nucleotide sequences. The tree was constructed using the Maximum Likelihood method with the Kimura 2-parameter model (+G, +I) in MEGA X (1000 bootstrap replicates). Bootstrap support values are shown next to the branches. The filled circle (●) and red arrow indicate the genome of KlebP 144 (PV528436), which contains the gene of DepKP144ΔC. Scale bar: 0.020 substitutions per site.

To determine the phylogenetic position of DepKP144ΔC among known phage-encoded depolymerases, a sequence-based analysis was performed using homologous sequences retrieved from the NCBI GenBank database. Multiple sequence alignment and tree reconstruction revealed that DepKP144ΔC clusters within a coherent group of depolymerases encoded by *Klebsiella* phages. This clustering pattern suggests a close functional relationship and supports the assumption that the C-terminal region of DepKP144 could determine its substrate specificity. Such domain-level plasticity likely reflects evolutionary adaptation to the structural diversity of *Klebsiella* capsular polysaccharides (Figure 1).

**Figure 2:**
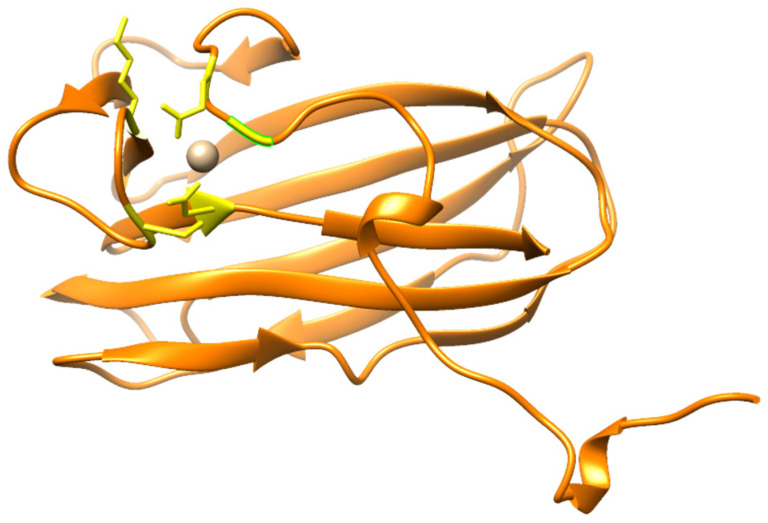
Ribbon representation of the predicted 3D structure of DepKP144ΔC in complex with Ca^2+^ ion. The putative Ca-binding site of DepKP144ΔC; Gly25, Glu27, Ser54, Lys57, and Asp163 are shown in a stick representation and marked yellow. The molecular coordinates of the predicted 3D structure of DepKP144ΔC were rendered using UCSF Chimera molecular visualizer, version 1.15.

The three-dimensional structure of DepKP144ΔC was predicted using AlphaFold3 v3.0.0 [20] (https://alphafoldserver.com/, accessed on 10 January 2026). The model reveals a compact and stable β-sandwich fold, specifically a jelly-roll topology. Analysis with the COFACTOR meta-server (accessed on 5 February 2026), which predicts protein–ligand binding sites, indicated structural similarity to proteins with hydrolase activity. The highest structural similarity (TM-score > 0.74) was observed with the enzymatically active C-terminal domain of the bacteriophage CBA120 tailspike protein (PDB: 5W6H), the carbohydrate-binding motif PsCBM61 (Arg1132-Pro1286) of *Paenibacillus* sp. 598K α-1,6-glucosyltransferase (Ps6TG31A, PDB: 5X7O), and the Family 16 Carbohydrate-Binding Module from *Thermoanaerobacterium polysaccharolyticum* ManA (PDB: 2ZEW). The ion-binding site of DepKP144ΔC was predicted using COFACTOR. This prediction indicated that the Ca ion (Ca^2+^) probably is coordinated with Gly25, Glu27, Ser54, Lys57, and Asp163 (Figure 2).

### 2.2. Cloning, Expression, and Purification of Recombinant Depolymerase Protein DepKP144ΔC

**Figure 3:**
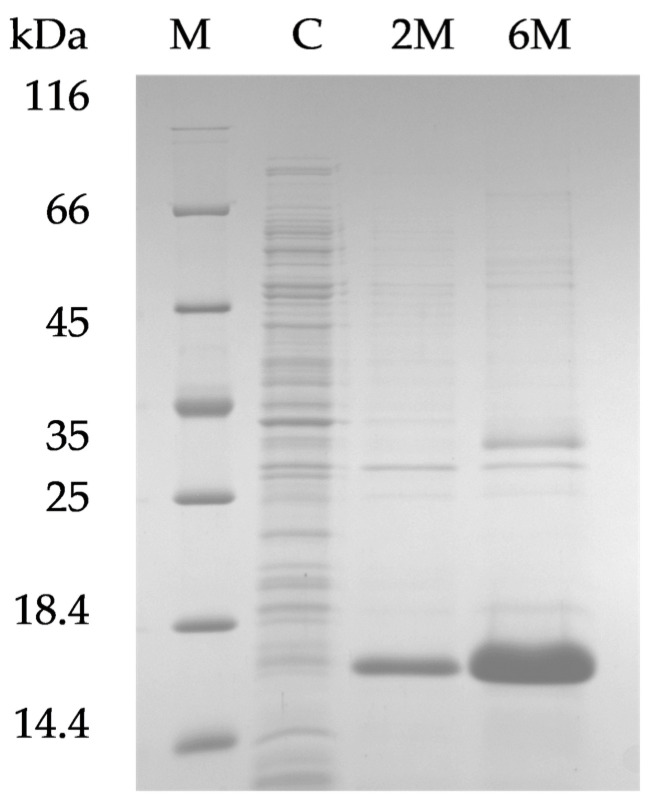
SDS-PAGE of lysates of *E. coli* BL21(DE3)/pET-28_ DepKP144ΔC. C—cytoplasmic fractions; 2 M—inclusion body fraction dissolved in 50 mM Tris-HCl (pH 8.0), containing 2 M urea; 6 M—inclusion body fraction dissolved in 50 mM Tris-HCl (pH 8.0), containing 6 M urea; M—molecular weight protein marker 26,610 (14.4–116 kDa) (Thermo Fisher Scientific, Waltham, MA, USA).

The gene encoding the putative capsular depolymerase DepKP144ΔC was successfully amplified from the genome of the isolated *K. pneumoniae* bacteriophage and cloned into a T7-based expression vector pET28a. *E. coli* BL21 (DE3) cells were transformed with the constructed plasmid, pET28_DepKP144ΔC, to produce the recombinant protein DepKP144ΔC. To improve the solubility of the recombinant protein, various cultivation conditions were tested. However, under all conditions tested, DepKP144ΔC was exclusively localized to the insoluble fraction and accumulated in the inclusion bodies (Figure 3).

**Figure 4:**
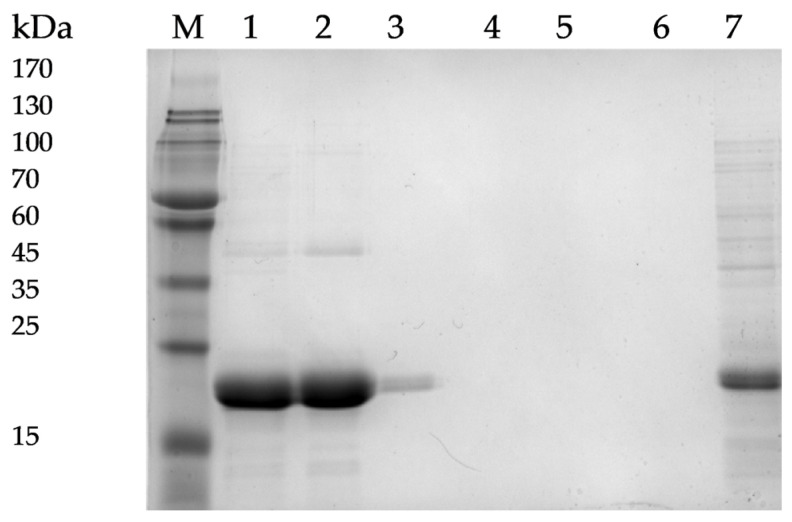
SDS-PAGE of purified pET-28_DepKP144ΔC. 1—elution with wash buffer containing 50 mM imidazole; 2—elution with wash buffer containing 100 mM imidazole; 3—elution with wash buffer containing 200 mM imidazole; 4—elution with wash buffer containing 500 mM imidazole; 5—elution with wash buffer containing 0.1 M EDTA; 6—elution with 50 mM NaOH, 1 M NaCl; 7—cell lysate before loading onto Ni-NTA agarose. M—Protein Ladder RAV11 (Biolabmix, Novosibirsk, Russia).

The protein was, therefore, purified from inclusion bodies under denaturing conditions using Ni–NTA agarose chromatography. After purification, DepKP144ΔC was refolded on-column or during dialysis using a series of buffers with gradually decreasing urea concentrations. The molecular weight of the purified protein was approximately 18 kDa, as estimated by SDS-PAGE, which is consistent with the predicted molecular weight. The presence of the C-terminal polyhistidine tag was confirmed by Western blotting using monoclonal anti-His antibodies (mAb.His1, Biosan, Novosibirsk, Russia). The purity of DepKP144ΔC after refolding and dialysis was estimated to be ~95%, as judged by densitometric analysis of Coomassie-stained SDS-PAGE gels (Figure 4).

The final yield of purified DepKP144ΔC was approximately 20 mg per liter of bacterial culture. The purified protein was stored at 4 °C in S-buffer (50 mM Tris–HCl, pH 7.5, 300 mM NaCl) at a concentration of at least 1 mg/mL.

### 2.3. Analysis of Enzymatic Activity of DepKP144ΔC Against K. pneumoniae Using the Diffusion Assay

**Figure 5:**
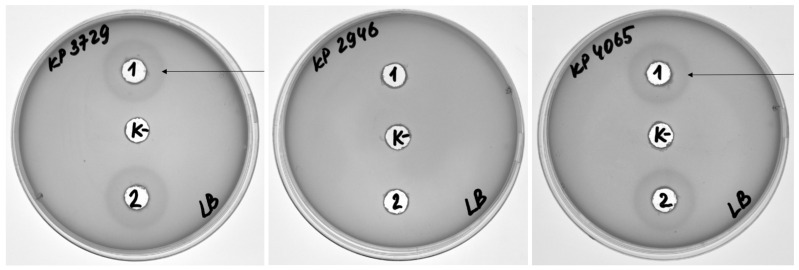
Hydrolytic activity of the DepKP144 ΔC protein against *K. pneumoniae* K2-type strains, determined using a diffusion test. A—example of the diffusion test; B—results of the protein activity study against the tested strains. K—negative control R-buffer (50 mM Tris-HCl; pH 8.0). The halo around the wells (anti-*Klebsiella* activity of the protein) is indicated by an arrow.

The enzymatic activity of DepKP144ΔC was assessed against clinical *K. pneumoniae* isolates obtained from the Culture Collection of Extremophilic Microorganisms and Type Cultures (CEMTC), ICBFM SB RAS, using the diffusion assay. It was demonstrated that DepKP144ΔC has enzymatic activity against 30 of 35 strains of *K. pneumoniae* K2-type, in contrast to parental phage KlebP_144, which could lyse only 19 of 35 strains of *K. pneumoniae* K2-type. The investigated panel of *K. pneumoniae* included both phage-susceptible and phage-resistant isolates, encompassing both mucoid and hypermucoid, as well as antibiotic-sensitive and multidrug-resistant (MDR) variants (Figure 5, Table 1 and Table S1).

**Figure 6:**
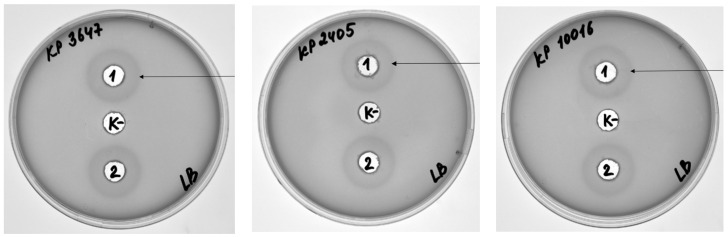
Hydrolytic activity of the DepKP144 ΔC protein against *K. pneumoniae* K1-type strains, determined using a diffusion test. A—example of the diffusion test; B—results of the protein activity study against the tested strains. K—negative control R-buffer (50 mM Tris-HCl; pH 8.0). The halo around the wells (anti-*Klebsiella* activity of the protein) is indicated by an arrow.

Following the same approach, the activity of DepKP144ΔC was evaluated against 19 clinical isolates of *K. pneumoniae* belonging to the K1 capsular type. Notably, the phage KlebP_144 is incapable of infecting K1-type *K. pneumoniae*. DepKP144ΔC demonstrated clear enzymatic activity against all 19 tested K1 isolates (Figure 6, Table 2 and Table S1).

**Figure 7:**
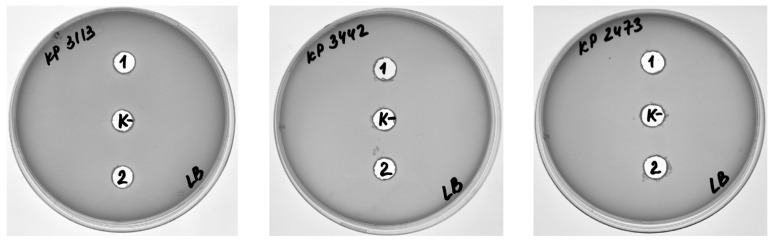
Hydrolytic activity of the DepKP144 ΔC protein against *K. pneumoniae* K9, K16, K17, K22, K35, K49, K51, K57, K63, and K108 strains, determined using a diffusion test. A—example of the diffusion test; B—results of the protein activity study against the tested strains. K—negative control R-buffer (50 mM Tris-HCl; pH 8.0).

Given that DepKP144ΔC exhibited activity toward both K1 and K2 capsular types, its substrate specificity was further examined using 10 additional *K. pneumoniae* isolates representing other capsular types (K9, K16, K17, K22, K35, K49, K51, K57, K63, and K108). No depolymerizing activity was detected against any of these strains (Figure 7, Table 3 and Table S1). Thus, DepKP144ΔC demonstrates a pronounced specificity toward *K. pneumoniae* strains of the K1 and K2 capsular types, with no detectable activity against isolates representing other capsular serotypes.

### 2.4. Analysis of Antibacterial Activity of DepKP144ΔC Against Planktonic Cells of K. pneumoniae In Vitro

**Figure 8:**
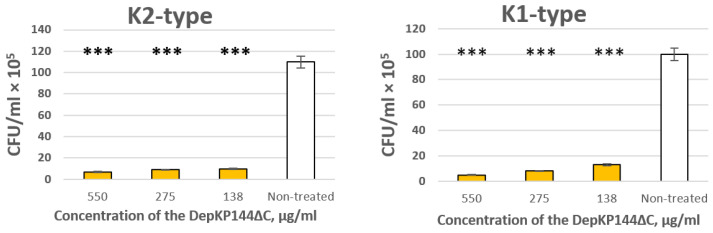
Antibacterial activity of DepKP144ΔC against *K. pneumoniae* strains CEMTC 9596 (K1-type) and 2067 (K2-type). DepKP144ΔC was added in concentrations of 550, 275, and 138 µg/mL to cells of *K. pneumoniae* and was incubated for two hours before viable colony counting. Cell cultures with R-buffer were used as controls. Experiments were performed in triplicate. *** *p* < 0.01.

The antibacterial activity of DepKP144ΔC was evaluated against *K. pneumoniae* strains CEMTC 9596 (K1-type) and CEMTC 2067 (K2-type) using a serial dilution assay. DepKP144ΔC exhibited concentration-dependent activity against both capsular types. Treatment with 550 µg/mL reduced the bacterial titer of the K1-type strain by 15-fold relative to untreated controls; at 275 µg/mL and 138 µg/mL, reductions of 12-fold and 11-fold were observed, respectively. For the K2-type strain, the same concentrations yielded 20-, 12-, and 8-fold reductions in bacterial titer. At all tested concentrations, the differences between treated and control groups were statistically significant (*p* < 0.01, one-way ANOVA; Figure 8). These findings are consistent with the established mechanism of phage-encoded capsule depolymerases, which attenuate bacterial virulence by degrading the CPS layer and thereby increasing susceptibility to complement-mediated killing and phagocytic clearance [21,22].

### 2.5. Antibiofilm Activity of DepKP144ΔC Against K. pneumoniae

**Figure 9:**
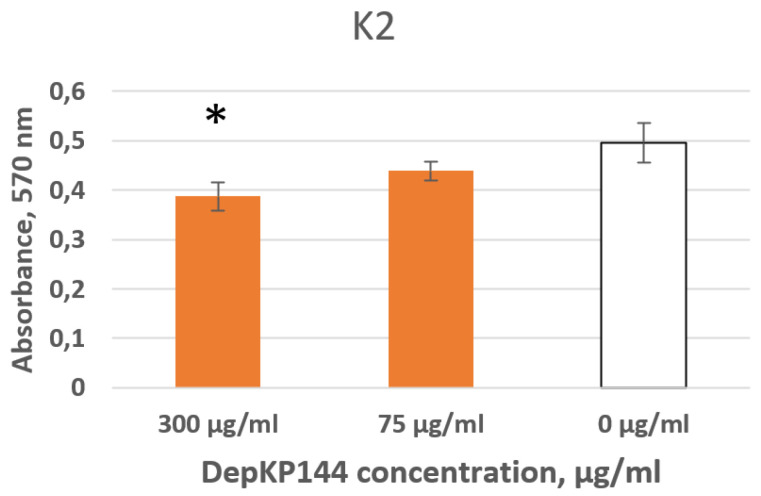
Inhibitory activity of DepKP144ΔC on biofilm formation by the mucoid *K. pneumoniae strain* CEMTC 2574 (K2-type). DepKP144ΔC was added at concentrations of 300 and 75 µg/mL to cells of *K. pneumoniae*, followed by incubation for two days. Biofilms were then stained with crystal violet. Cells treated with R buffer served as controls. Experiments were performed in triplicate. * *p* < 0.05.

Treatment with the depolymerase fragment DepKP144ΔC affected biofilm formation in a strain-dependent manner. In particular, a reproducible reduction in total biofilm formation was observed for *K. pneumoniae* strain CEMTC 2574 (K2). At a concentration of 300 μg/mL, DepKP144ΔC decreased biofilm formation by approximately 25%, whereas a lower concentration of 75 μg/mL resulted in a reduction of about 12.5%. No complete suppression of biofilm development was detected under the tested conditions. This partial inhibitory effect suggests that while CPS removal impairs the early adhesion steps, it does not fully abrogate biofilm formation in this strain, possibly due to CPS-independent biofilm stabilisation mechanisms. Further studies using isogenic capsule-deficient mutants would be required to delineate the specific contribution of CPS to biofilm formation in this isolate. (Figure 9).

### 2.6. In Vivo Evaluation of DepKP144ΔC Depolymerase Activity in a Murine Systemic Klebsiella Infection Model

The in vivo antibacterial activity of DepKP144ΔC was assessed using a murine systemic Klebsiella infection model. Animals were randomly assigned to the following experimental groups: intact control (uninfected, untreated); (i) control of protein (uninfected with K2-type *K. pneumoniae*, DepKP144ΔC-treated groups); (ii) infection control (infected, untreated); (iii) negative protein control, receiving an unrelated protein NS1 (20 µg or 100 µg per mouse); (iv) phage-treated group, infected and treated with phage KlebP_144 (10^9^ PFU per mouse); (v) DepKP144ΔC-treated groups, infected and treated with DepKP144ΔC alone (36 µg or 180 µg per mouse). During the first three days postinfection, all infected animals, irrespective of the treatment regimen, developed pronounced clinical signs of systemic infection, including reduced food intake, dehydration, increased muscle tone, and a decrease in body weight of approximately 1–2 g relative to baseline. An exception was observed in the phage-treated group infected with *K. pneumoniae* CEMTC 2067 (capsular type K2), where animals began to recover body weight as early as day two postinfection. From day four onward, surviving mice in the remaining experimental groups gradually regained activity and demonstrated a net weight gain of 2–3 g compared to their initial values.

All animals in the negative protein control groups receiving NS1 reached the humane endpoint within 24–48 h after infection, confirming the absence of nonspecific protective effects. In contrast, mice in the intact control group remained clinically healthy throughout the observation period, showing stable weight gain and normal behavior. Bacteriological analysis of tissue homogenates collected from surviving animals in the experimental and intact control groups revealed no detectable bacterial growth, indicating effective clearance of *K. pneumoniae.*

**Figure 10:**
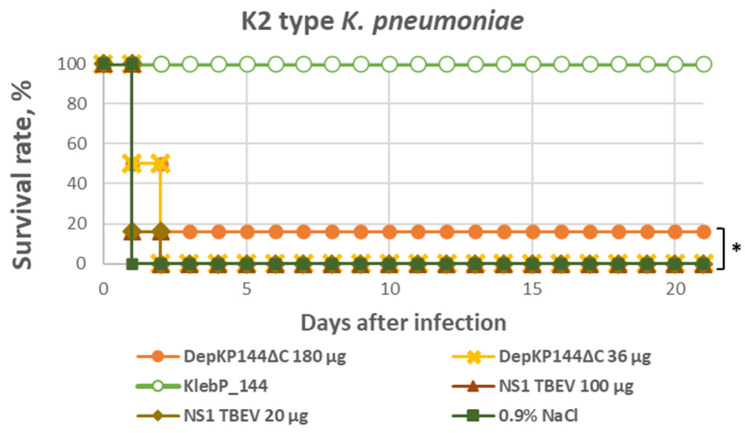
Efficacy of DepKP144ΔC in post-exposure prophylaxis after infection by *K. pneumoniae strain* CEMTC 2067 (K2-type). Female BALB/c mice (18 g) were treated i.p. with DepKP144ΔC, bacteriophage KlebP_144 or NS1 protein at the indicated doses 2 h after injection of 1 × 10^8^ colony-forming units of *K. pneumoniae strain* CEMTC 2067 (K2-type). * *p* < 0.05 (log-rank test).

Treatment outcomes differed markedly between experimental regimens. Administration of bacteriophage KlebP_144 to mice infected with its host strain *K. pneumoniae* CEMTC 2067 (K2 type) resulted in 100% survival, reflecting the pronounced lytic activity of the phage in vivo. In contrast, animals infected with the same strain but not receiving any therapy did not survive. Administration of DepKP144ΔC provided a measurable, albeit limited, protective effect. At a dose of 180 µg per mouse, survival reached approximately 25% in mice infected with the *K. pneumoniae* K2-type strain. Notably, decreasing the dose to 36 µg per mouse completely abolished the protective effect, leading to 100% mortality. This dose-dependent loss of efficacy suggests a narrow therapeutic window for DepKP144ΔC under the tested conditions (Figure 10).

## 3. Discussion

Hypervirulent *K. pneumoniae* strains, particularly those belonging to serotypes K1 and K2, exhibit higher virulence and cause higher mortality rates compared to other strains of this species [23,24,25]. Depolymerases—bacteriophage-derived proteins—are capable of degrading capsular polysaccharide (CPS), a key virulence factor, and have demonstrated pronounced therapeutic potential against *K. pneumoniae* infections [21,22].

Phage-encoded polysaccharide depolymerases typically have a modular three-domain structure [18]. The N-terminal domain ensures virion incorporation and proper surface orientation. The central β-helical trimeric domain recognizes and degrades exopolysaccharides of Gram-negative bacteria [26]. The variable C-terminal domain binds capsular or extracellular polysaccharides or assists in folding and trimerization [27]. These regions determine substrate specificity and selectivity toward *K. pneumoniae* capsular types.

Bioinformatic analysis of the DepKP144ΔC amino acid sequence revealed its phylogenetic similarity to the C-terminal domains of other *Klebsiella* bacteriophage depolymerases, with which it clusters to form a distinct clade. Structural analysis of DepKP144ΔC demonstrated a high degree of similarity to the carbohydrate-binding modules (CBMs) of various glucosyltransferases capable of cleaving complex sugars. For instance, DepKP144ΔC shows significant similarity to the PsCBM61 domain of the *Paenibacillus* sp. 598K alpha-1,6-glucosyltransferase [26]. Although the precise function of the PsCBM61 domain is not fully established, it is known to bind both α-1,4-glucan and α-1,6-glucan, which are products generated from the enzymatic cleavage of the substrate by the *Paenibacillus* sp. 598K alpha-1,6-glucosyltransferase [26]. Therefore, the role of DepKP144ΔC within the fiber protein of bacteriophage Kleb_144 likely extends beyond initial receptor binding during phage adsorption to the target cell. It may also be involved in binding the intermediate products resulting from the enzymatic degradation of the *K. pneumoniae* cell wall exopolysaccharides.

Under in vitro conditions, DepKP144ΔC exhibited polysaccharide-depolymerizing activity toward planktonic cultures of *K. pneumoniae* K1 and K2. These results suggest that DepKP144ΔC can function not only as an auxiliary (adjuvant) molecule but also as an independent antibacterial agent. Beyond its activity against planktonic cells, the capacity of DepKP144ΔC to interfere with biofilm formation further expands its potential translational relevance. Capsular polysaccharide contributes to early adhesion and stabilization of developing microcolonies, and its enzymatic removal may disrupt the initial stages of biofilm maturation. However, the partial biofilm inhibition suggests that additional extracellular matrix components, including proteins and extracellular DNA, likely sustain structural integrity once the biofilm is established. The demonstrated efficacy of DepKP144ΔC in vivo confirmed the potential of depolymerases as a promising therapeutic agent. These findings indicate that depolymerases may be most effective when applied during early infection stages or in combination with complementary therapeutic agents.

It is important to emphasize that depolymerases do not exert direct bactericidal effects in the classical sense [27]. Rather, enzymatic removal of the capsule exposes underlying outer membrane structures, thereby increasing bacterial susceptibility to complement-mediated lysis, opsonization, and phagocytic clearance [18]. The ability of DepKP144ΔC to target both K1 and K2 capsules distinguishes it from the majority of Klebsiella phage depolymerases, which are typically capsular serotype-restricted. Given the structural divergence of K1 and K2 CPS repeating units, such cross-reactivity suggests either recognition of a conserved glycan motif or a broader substrate-binding interface capable of accommodating structurally distinct polysaccharides. Such structural adaptability could represent an evolutionary advantage, enabling bacteriophages to maintain infectivity within heterogeneous bacterial populations where multiple hypervirulent lineages co-circulate.

This study has several limitations. First, the precise chemical bonds cleaved by DepKP144ΔC were not determined. Bioinformatic analysis enabled the prediction of a putative glycan-binding site; however, the exact mechanism of glycosidic bond cleavage remains to be elucidated. Second, although the safety and efficacy of DepKP144ΔC were demonstrated in a systemic *Klebsiella* Infection, further validation at the clinical level is required. Finally, the interaction between phage depolymerases and host bacteria is complex. A comprehensive understanding of the bactericidal effects of phage depolymerases and their fragments will require additional studies aimed at elucidating their detailed mechanisms of action.

## 4. Materials and Methods

### 4.1. Bacterial Strains, Phage, and Growth Conditions

All bacterial strains and bacteriophages used in this study are listed below. *K. pneumoniae* isolates of capsular types K1, K2, and other serotypes were obtained from the CEMTC. In total, 19 K1-type and 35 K2-type *K. pneumoniae* isolates, as well as 10 strains representing other capsular types (K9, K16, K17, K22, K35, K49, K51, K57, K63, and K108), were included in this work. Detailed strain information is provided in Supplementary Table S1.

The following *E. coli* strains were used for cloning and protein expression: *E. coli* DH5α (F– endA1 glnV44 thi1 recA1 relA1 gyrA96 deoR nupG purB20 φ80dlacZΔM15 Δ(lacZYA-argF)U169, hsdR17(rK–mK+), λ–) and *E. coli* BL21(DE3) (F– ompT gal dcm lon hsdSB(rB–mB–) λ(DE3 [lacI lacUV5-T7p07 ind1 sam7 nin5]) [malB+] K-12 (λS)).

Bacterial cultures were maintained in lysogeny broth (LB) medium.

Bacteriophage *K. pneumoniae* KlebP_144, previously isolated and characterized at the Institute of Chemical Biology and Fundamental Medicine (ICBFM SB RAS, Novosibirsk, Russia), was used in this study. The phage genome sequence is available in the NCBI GenBank Database (accession no. PV528436).

### 4.2. Animals

All animal experiments were performed in accordance with institutional and national guidelines for the care and use of laboratory animals and were approved by the Institutional Animal Care and Use Committee of the Institute of Chemical Biology and Fundamental Medicine, Siberian Branch of the Russian Academy of Sciences. Female BALB/c mice, 6–8 weeks of age and weighing 18–22 g, were obtained from the certified breeding facility (Vector State Research Center of Virology and Biotechnology VECTOR in Novosibirsk) and housed under specific conditions with ad libitum access to food and water. Animals were acclimatized for at least 7 days before infection and were randomly assigned to experimental groups.

### 4.3. Phylogenetic and In Silico Structural Analysis of the C-Terminal Domain of Depolymerase DepKP144

Phylogenetic relationships were inferred using the Maximum Likelihood approach implemented in MEGA X, based on aligned nucleotide sequences and applying the Kimura two-parameter substitution model. Tree robustness was assessed by bootstrap analysis, with evolutionary rate heterogeneity among sites modeled using a discrete gamma distribution and a proportion of invariable sites, after excluding poorly aligned positions by partial deletion.

The C-terminal domain of the depolymerase DepKP144 (DepKP144ΔC, GenBank: XRR08247.1) was analyzed using the InterPro software package v.108.0 (https://www.ebi.ac.uk/interpro/search/sequence/, accessed on 21 January 2026), HHpred tool (https://toolkit.tuebingen.mpg.de/tools/hhpred, accessed on 21 January 2026), and COFACTOR function prediction server (https://aideepmed.com/COFACTOR/, accessed on 19 May 2025) [28]. Protein solubility of DepKP144ΔC was predicted with the Protein-sol [29] (https://protein-sol.manchester.ac.uk, accessed on 15 August 2024) and SoluProt v1.0 [30] (https://loschmidt.chemi.muni.cz/soluprot, accessed on 15 August 2024). The three-dimensional structure models of the DepKP144ΔC were generated using AlphaFold2 v2.3.1 [31] (https://colab.research.google.com/github/sokrypton/ColabFold/blob/main/AlphaFold2.ipynb, accessed on 21 May 2025) and AlphaFold3 v3.0.0 (accessed on 19 January 2026) [20]. Structural visualization was performed using UCSF Chimera molecular visualizer, version 1.15 [32].

### 4.4. Protein Cloning, Expression, and Purification

The gene encoding the DepKP144ΔC protein was amplified by PCR using Kleb_P144 genomic DNA as a template and the primer pair DepKP144ΔC_U (5′ GGGCTCCATGGCGTGTACATACGACATTGAATCGGG 3′)/DepKP144ΔC_L (5′ CCCTTGGATCCGTCAAGAAGTTTACGATAACGTCGTCAAGG 3′). The expression vector pET28a was digested with *Nco*I and *Bam*HI restriction endonucleases (Sibenzyme, Novosibirsk, Russia). The PCR product was subsequently digested with the same enzymes and ligated into the linearized pET28a backbone. The resulting construct, designated pET28a_DepKP144ΔC, was used to transform *E. coli* XL1-Blue competent cells. Transformants were selected on LB agar plates supplemented with 50 µg/mL kanamycin and incubated overnight at 37 °C. Colony PCR with the pET28-SeqU and pET28-SeqL primers was performed to screen for clones harboring the correct insert. Positive clones were verified by Sanger sequencing on a 3500 Genetic Analyzer (Applied Biosystems, Foster City, CA, USA).

To optimize expression, the pET28a_DepKP144ΔC plasmid was transformed into *E. coli* BL21(DE3) cells. Transformants were selected on LB agar plates containing 25 µg/mL kanamycin and grown overnight at 37 °C. For protein production, a single colony was used to inoculate a liquid LB medium with the same antibiotic concentration. The culture was incubated at 37 °C with shaking until the optical density at 600 nm (OD_600_) reached 0.6–0.7. Protein expression was then induced by adding isopropyl β-D-1-thiogalactopyranoside (IPTG) at final concentrations ranging from 0.1 to 1 mM. To determine optimal conditions, induced cultures were grown overnight at different temperatures (12, 20, 30, and 37 °C) with shaking at 150 rpm. Cells were harvested by centrifugation (6000× *g*, 10 min), resuspended in 50 mM Tris-HCl (pH 8.0), and lysed by sonication. The insoluble fraction was dissolved in 50 mM Tris-HCl (pH 8.0) containing 2 M or 6 M urea and sonicated. The expression levels and cellular localization (soluble vs. insoluble fraction) of the recombinant protein were analyzed by 12.5% SDS-PAGE.

The recombinant protein DepKP144ΔC was purified under denaturing conditions using Ni-NTA agarose resin. A column was packed with the resin (Sigma-Aldrich, St. Louis, MO, USA) and equilibrated with 10 column volumes of binding/wash buffer (50 mM NaH_2_PO_4_, 300 mM NaCl, 10 mM imidazole, 6 M urea, pH 8.0). The clarified lysate containing the target protein was loaded onto the column at a flow rate of 1 mL/min. After sample application, the column was washed with the same buffer to remove unbound and weakly associated proteins. Bound proteins were then eluted stepwise using buffers with increasing imidazole concentrations (50, 100, 200, and 500 mM), prepared in the same base solution (50 mM NaH_2_PO_4_, 300 mM NaCl, 6 M urea, pH 8.0). Target protein fractions were collected and analyzed.

Eluted fractions were analyzed by 12% SDS-PAGE, and gels were visualized with a GelDoc Go imaging system (Bio-Rad, Hercules, CA, USA). Fractions containing the target protein at the expected molecular weight and with sufficient purity were pooled. The purified protein, obtained under denaturing conditions, was refolded by stepwise dialysis at 4 °C. Dialysis was performed sequentially against the following buffers: (i) buffer 1 (1.5 M urea, 50 mM imidazole, 300 mM NaCl, 200 mM sucrose, 0.1% Triton X-100, pH 7.4); (ii) buffer 2 (0.5 M urea, 10 mM imidazole, 300 mM NaCl, 200 mM sucrose, 0.1% Triton X-100, pH 7.4); (iii) buffer 3 (50 mM Tris–HCl, 300 mM NaCl, 200 mM sucrose, pH 7.4). Each dialysis step was performed for 12–16 h at +4 °C. The refolded protein solutions were sterilized by filtration through 0.22 µm pore filters and stored at 4 °C. Protein concentrations were determined using a Qubit 4.0 fluorometer (Thermo Fisher Scientific, Waltham, MA, USA).

### 4.5. Anti-Klebsiella Activity Assay In Vitro

The ability of the depolymerase fragment DepKP144ΔC to lyse *Klebsiella* cells was assessed by counting the number of colony-forming units (CFU) after incubating *Klebsiella* cells with DepKP144ΔC using a previously described protocol, with brief modifications [33]. Each *Klebsiella* strain was freshly cultivated to the exponential phase (OD600 = 0.5) and centrifuged at 4000× *g* for five minutes. The harvested cells were washed and suspended in R-buffer (50 mM Tris-HCl; pH 8.0); the cell suspension was diluted to a titer of 10^6^ CFU/mL. Then, DepKP144ΔC was diluted in R buffer at various concentrations. Cells without DepKP144ΔC were used as a control. A total of 100 µL of cell suspension was mixed with serial dilutions of DepKP144ΔC in 96-well plates and incubated at 37 °C for 2 h. Aliquots of suspensions were plated on LB agar plates; colonies were counted the next day.

### 4.6. In Vitro Biofilm Assay

The effect of the depolymerase fragment DepKP144ΔC on biofilm formation and stability of *K. pneumoniae* was assessed in vitro using a microtiter plate-based assay combined with crystal violet staining. A panel of clinical *K. pneumoniae* isolates representing different capsular types was included in the analysis, comprising strains 2754 (K2) and 2894 (K1).

To evaluate inhibition of biofilm formation, bacterial cultures were grown to the exponential phase and adjusted to an optical density of OD_600_ = 0.1, followed by a 1:100 dilution in fresh LB medium. Aliquots of 180 μL were dispensed into sterile flat-bottom 96-well microplates, after which 20 μL of DepKP144ΔC solution in S-buffer was added to achieve final concentrations of 75 or 300 μg/mL. Control wells received an equal volume of phosphate-buffered saline (PBS). Plates were incubated statically at 37 °C for 24 h.

For assessment of biofilm disruption, a modified protocol was employed. Log-phase cultures were adjusted to OD_600_ = 0.1, diluted 100-fold, and transferred to microplate wells (200 μL per well). After incubation at 37 °C for 48 h to allow mature biofilm formation, planktonic cells were removed, and wells were washed three times with PBS. Subsequently, 200 μL of DepKP144ΔC solution at the indicated concentrations or PBS alone was added, followed by an additional incubation for 6 h at 37 °C.

Quantification of biofilm biomass was performed using crystal violet staining. After treatment, wells were washed with PBS and air-dried to fix biofilms. Fixed biofilms were stained with 0.1% (w/v) crystal violet solution for 20 min, rinsed thoroughly with water three times, and dried. The bound dye was solubilized with 96% ethanol, and absorbance was measured at 570 nm. All experiments were conducted in at least three independent biological replicates, each with three technical replicates.

### 4.7. Murine Model of Systemic Klebsiella Infection

The in vivo therapeutic potential of DepKP144ΔC was evaluated in a murine model of systemic *K. pneumoniae* infection, adapted from a previously established protocol [32]. Briefly, *K. pneumoniae* strain CEMTC 2067 was cultured overnight in LB medium at 37 °C. Bacterial cells were harvested by centrifugation, washed twice with sterile 0.9% NaCl, and resuspended to an optical density at 600 nm (OD_600_) corresponding to the desired concentration. Six-week-old female BALB/c mice were infected intraperitoneally (i.p.) with 0.1 mL of the bacterial suspension containing 1 × 10^8^ colony-forming units (CFU). One hour postinfection, mice were treated with a single intraperitoneal injection (0.5 mL) of one of the following: DepKP144ΔC at a dose of 36 µg or 180 µg per mouse, wild-type bacteriophage KlebP_144 (1 × 10^9^ PFU per mouse), or the control protein NS1 (20 µg or 100 µg per mouse) as an unrelated protein control [34]. The control group received an equivalent volume of saline. Each experimental and control group consisted of six mice (*n* = 6). Mice were monitored daily for 14 days, with body weight recorded daily as a clinical parameter. The primary endpoint was survival rate.

### 4.8. Statistical Analysis

Statistical analysis of the serial dilution assay results obtained from the control and experimental groups was performed using one-way ANOVA. A *p*-value < 0.01 was considered to indicate statistically significant differences in bacterial survival between groups.

Survival data from the in vivo mouse experiments were analyzed using the log-rank (Mantel–Cox) test, a non-parametric method that compares survival distributions between experimental groups over the entire observation period without assuming a particular distribution of survival times. Differences were regarded as statistically significant at *p* < 0.05 or *p* < 0.01. All log-rank and ANOVA calculations were performed using GraphPad Prism version 8.0 (GraphPad Software Inc., Boston, MA, USA).

## Figures and Tables

**Table 1:** Hydrolytic activity of DepKP144ΔC against *K. pneumoniae* K2-type clinical isolates and their sensitivity to bacteriophage KlebP_144. (+)—susceptible strain, (-)—non-susceptible strain.

CEMTC	Antibiotic Resistance	Hydrolytic Activity of the DepKP144	Sensitivity to Bacteriophage KlebP_144
**2067**	R	+	+
**2071**	MDR	+	+
**2291**	S	+	+
**2548**	MDR	+	+
**2574**	XDR	-	-
**2576**	MDR	-	-
**2728**	MDR	-	-
**2945**	MDR	-	-
**2946**	MDR	-	-
**3339**		+	-
**3521**	MDR	+	+
**3522**	MDR	+	+
**3729**	R	+	+
**3772**	MDR	+	-
**4065**	MDR	+	+
**4069**	MDR	+	-
**4087**	R	+	+
**4090**	R	+	+
**4117**	R	+	+
**4124**	S	+	-
**4128**	S	+	-
**4162**	S	+	-
**4163**	R	+	-
**4169**	S	+	-
**5232**	S	+	+
**5234**	S	+	+
**6824**	MDR	+	-
**6846**	MDR	+	-
**6851**	S	+	-
**9609**	R	+	-
**9874**	MDR	+	-
**10083**	S	+	-
**10125**	S	+	-
**10126**	S	+	-

**Table 2:** Hydrolytic activity of DepKP144ΔC against *K. pneumoniae* K1-type clinical isolates and their sensitivity to bacteriophage KlebP_144. (+)—susceptible strain, (-)—non-susceptible strain.

CEMTC	Antibiotic Resistance	Hydrolytic Activity of the DepKP144	Sensitivity to Bacteriophage KlebP_144
**2405**	S	+	-
**2810**	R	+	-
**2894**	MDR	+	-
**3632**	R	+	-
**3646**	R	+	-
**3647**	R	+	-
**4160**	S	+	-
**6604**	S	+	-
**8557**	S	+	-
**9080**	S	+	-
**9478**	S	+	-
**9479**	S	+	-
**9596**	S	+	-
**9597**	S	+	-
**10016**	S	+	-
**10160**	S	+	-
**10162**	S	+	-
**10634**	S	+	-
**10637**	S	+	-

**Table 3:** Hydrolytic activity of DepKP144ΔC against *K. pneumoniae* isolates representing other capsular types (K9, K16, K17, K22, K35, K49, K51, K57, K63, K108) and their sensitivity to bacteriophage KlebP_144. (-)—non-susceptible strain.

CEMTC	Antibiotic Resistance	Hydrolytic Activity of the DepKP144	Sensitivity to Bacteriophage KlebP_144
**K9-type *K. pneumonia***			
**2826**	R	-	-
**K16-type *K. pneumoniae***			
**3113**	R	-	-
**K17-type *K. pneumoniae***			
**3838**	MDR	-	-
**K22-type *K. pneumoniae***			
**2573**	R	-	-
**K35-type *K. pneumoniae***			
**1751**	R	-	-
**K49-type *K. pneumoniae***			
**2394**	S	-	-
**K51-type *K. pneumoniae***			
**3442**	MDR	-	-
**K57-type *K. pneumoniae***			
**4194**	R	-	-
**K63-type *K. pneumoniae***			
**1609**	R	-	-
**K108-type *K. pneumoniae***			
**2646**	S		

## Data Availability

Data are contained within the article and Supplementary Materials.

## Supplementary Materials

The following supporting information can be downloaded at: https://www.mdpi.com/article/10.3390/ijms27093883/s1.

## Abbreviations

The following abbreviations are used in this manuscript:

LCRKP	Carbapenem-resistant *K. pneumoniae*
K-types	Capsular types
LB	Luria–Bertani
CFU	Colony-forming units
EPS	Exopolysaccharide
LD50	Median lethal dose
CPS	Capsular polysaccharide
MDR	Multidrug-resistant
SDS-PAGE	Sodium dodecyl sulfate–polyacrylamide gel electrophoresis
PFU	Plaque-forming units
OD	Optical density
PCR	Polymerase chain reaction
IPTG	Isopropyl β-D-1-thiogalactopyranoside
EDTA	Ethylenediaminetetraacetic acid
ANOVA	Analysis of variance
NCBI	National Center for Biotechnology Information
PDB	Protein Data Bank
ICBFM SB RAS	Institute of Chemical Biology and Fundamental Medicine, Siberian Branch of the Russian Academy of Sciences
MDR	Multidrug resistance
R	Resistance
S	Sensitive
XDR	Extensively Drug Resistant
