# Supplementary material for: Characterization of the Fiber Protein C-Terminal Domain from Klebsiella pneumoniae Phage KlebP_144 and Evaluation of Its Anti-Capsular Activity
Source: Int J Mol Sci. 2026 Apr 27;27(9):3883. doi: 10.3390/ijms27093883 (PMC13163572; doi:10.3390/ijms27093883)
Supplement: Supplementary file 1 [file ijms-27-03883-s001.zip › Table S1.pdf]

Table S1. Characteristics of *K. pneumoniae* strains.

CEMTC	Sample source	Antibiotic resistance	Aminoglycosides	Macrolides	Lincosamides	Fluoroquinolones	Chloramphenicol	Sulphonamides	Other antibiotics	Antibiotic resistance
**K1-type *K. pneumoniae***										
2405	feces									S
2810	vaginal smear	SAM								R
**2894**	**surgical wound**	**AM, AMC, SAM/CAZ, FEP, CTX, CRO, CXM, CEC, CFM/ATM**				**NOR**			**F**	**MDR**
**3632**	**feces**					**CIP**	**C**			**R**
**3646**	**oral mucosa**					**LEV, CIP**	**C**			**R**
**3647**	**oral mucosa**	**AMC**								**R**
**4160**	**throat swab**									**S**
6604	phlegm									
8557	oral mucosa									S
9080	oral mucosa									
**9478**	**throat swab**									
**9479**	**throat swab**									
9596	nose swab									
**9597**	**nose swab**									
10016	nose swab									
10160	feces									
10162	throat swab									
10634	nose swab									
10637	nose swab									
**K2-type *K. pneumoniae***										
**2067**	**surgical wound**					**LEV, CIP**	**C**			**R**
2071	synovial fluid		CN			LEV, CIP	C			MDR
**2291**	**throat swab**									**S**

CEMTC	Sample source	Antibiotic resistance	Aminoglycosides	Macrolides	Lincosamides	Fluoroquinolones	Chloramphenicol	Sulphonamides	Other antibiotics	Antibiotic resistance
					K1-type *K. pneumoniae*					
2548	urine	AMC, SAM/CAZ/ATM	CN			LEV, CIP	C			MDR
2574	urine	AMC, SAM, CEC, CXM, CFM/CAZ/ATM	CN	CLR		LEV, CIP, GF	C	SXT		XDR
2576	urine	AMC, SAM/CAZ/ ATM	CN			LEV, CIP	C			MDR
2728	urine	AMC, SAM, TPZ/CAZ/ATM	CN			LEV, CIP	C			MDR
2945	urine	AMC, SAM/CAZ/ATM	CN			LEV, CIP	C			MDR
2946	urine	AMC, SAM, TPZ/CAZ/ATM	CN			LEV, CIP	C			MDR
3339	urine									
3521	urine	AMC, SAM, TPZ/MEM, IPM				LEV, CIP	C			MDR
3522	urine	AMC, SAM, TPZ				LEV, CIP	C			MDR
3729	oral mucosa					CIP				R
3772	hematoma	AMC, SAM, TPZ				LEV, CIP	C			MDR
**4065**	**throat swab**	**AMC, SAM, TIM/FOX, CAZ, FEP/MEM, IPM/ATM**	**AK**				**C**			**MDR**
4069	throat swab	AMC, SAM, TPZ/CAZ	AK			LEV, CIP				MDR
**4087**	**throat swab**	**AM, SAM**								**R**
**4090**	**throat swab**	**AM, SAM**								**R**
**4117**	**throat swab**	**AM, SAM**								**R**
**4124**	**feces**									**S**
4128	feces									S
**4162**	**throat swab**									**S**

CEMTC	Sample source	Antibiotic resistance	Aminoglycosides	Macrolides	Lincosamides	Fluoroquinolones	Chloramphenicol	Sulphonamides	Other antibiotics	Antibiotic resistance
K1-type *K. pneumoniae*										
**4163**	**throat swab**		**AK, CN**							**R**
4169	throat swab									S
5232	phlegm									S
5234	phlegm									S
6824	throat swab	AM, SAM, TPZ, TIM, AMC/FOX, FEP, CAZ/IPM, MEM/ATM	AK, CN			LEV, CIP		SXT		MDR
6846	feces	AM, SAM, TPZ, TIM, AMC/FEP, CAZ/ATM	AK, CN					SXT		MDR
6851	throat swab									
**9609**	**throat swab**	**TIM**								**R**
9874	wound	AM, SAM, AMC, TPZ/FEP, CAZ, CRO/ATM/IPM, MEM, DOR	AK, CN			LEV, CIP		SXT		MDR
10083	flush the ICU sink									
10125	flush the toilet									
10126	flush the toilet									
K9-type *K. pneumoniae*										
**2826**	**vaginal smear**	**AMC, SAM/CAZ/ATM**								**R**
K16-type *K. pneumoniae*										
**3113**	**feces**	**AMC, SAM, TPZ**								**R**
K17-type *K. pneumoniae*										
3838	feces	AMC, SAM, TPZ/CAZ/MEM, IPM/ATM	AK, CN			LEV, CIP				MDR
K22-type *K. pneumoniae*										

CEMTC	Sample source	Antibiotic resistance	Aminoglycosides	Macrolides	Lincosamides	Fluoroquinolones	Chloramphenicol	Sulphonamides	Other antibiotics	Antibiotic resistance
					**K1-type *K. pneumoniae***					
**2473**	**feces**	**TPZ/CAZ/ ATM**								**R**
					**K35-type *K. pneumoniae***					
1751	feces	TPZ				CIP				R
					**K49-type *K. pneumoniae***					
**2394**	**open drainage water**									
					**K51-type *K. pneumoniae***					
3442	throat swab	AMC, SAM, TPZ/CAZ/MEM/ATM	AK, CN			LEV, CIP	C			MDR
					**K57-type *K. pneumoniae***					
4194	throat swab		CN							R
					**K63-type *K. pneumoniae***					
1609	river water, bottom sediments	ATM								R
					**K108-type *K. pneumoniae***					
**2646**	**feces**									**S**

Abbreviations: MDR – Multi Drug Resistance; R – Resistance; S – Sensitive; AK – Amikacin; C - Chloramphenicol; CIP - Ciprofloxacin, CN – Gentamicin; DA – Clindamycin; FOX – Cefoxitin; E – Erytromycin; LEV – Levofloxacin; LNM – Lincomycin; P – Penicillin; SXT - Trimethoprim/sulfamethoxazole; TE – Tetracycline; VA – Vancomycin. Hypermuccoid strains of *K. pneumoniae* are highlighted in bold. The strains of *K. pneumoniae* that are sensitive to bacteriophage Kleb_P144 are highlighted in orange.
